# Kinetic and Structural Evidences on Human Prolidase Pathological Mutants Suggest Strategies for Enzyme Functional Rescue

**DOI:** 10.1371/journal.pone.0058792

**Published:** 2013-03-13

**Authors:** Roberta Besio, Roberta Gioia, Federica Cossu, Enrico Monzani, Stefania Nicolis, Lucia Cucca, Antonella Profumo, Luigi Casella, Ruggero Tenni, Martino Bolognesi, Antonio Rossi, Antonella Forlino

**Affiliations:** 1 Department of Molecular Medicine, Biochemistry Unit, University of Pavia, Pavia, Italy; 2 Department of BioSciences, CNR-IBF and CIMAINA, University of Milano, Milano, Italy; 3 Department of Chemistry, University of Pavia, Pavia, Italy; University of Edinburgh, United Kingdom

## Abstract

Prolidase is the only human enzyme responsible for the digestion of iminodipeptides containing proline or hydroxyproline at their C-terminal end, being a key player in extracellular matrix remodeling. Prolidase deficiency (PD) is an intractable loss of function disease, characterized by mutations in the prolidase gene. The exact causes of activity impairment in mutant prolidase are still unknown. We generated three recombinant prolidase forms, hRecProl-231delY, hRecProl-E412K and hRecProl-G448R, reproducing three mutations identified in homozygous PD patients. The enzymes showed very low catalytic efficiency, thermal instability and changes in protein conformation. No variation of Mn(II) cofactor affinity was detected for hRecProl-E412K; a compromised ability to bind the cofactor was found in hRecProl-231delY and Mn(II) was totally absent in hRecProl-G448R. Furthermore, local structure perturbations for all three mutants were predicted by *in silico* analysis. Our biochemical investigation of the three causative alleles identified in perturbed folding/instability, and in consequent partial prolidase degradation, the main reasons for enzyme inactivity. Based on the above considerations we were able to rescue part of the prolidase activity in patients’ fibroblasts through the induction of Heath Shock Proteins expression, hinting at new promising avenues for PD treatment.

## Introduction

Missense mutations are genetic alterations, resulting in the production of a protein with a single amino acid substitution, that are a common cause of a variety of heritable diseases [1]. The identification of the molecular defect is indeed a useful diagnostic tool, but alone it does not allow either deep understanding of the disease nor the development of appropriate therapies. To understand the cellular effects of a mutation, and to find the proper target for clinical intervention, a biochemical investigation is in fact always needed. Prolidase deficiency (OMIM 170100) is a loss of function disorder caused by missense mutations for about half of the characterized cases, and for which no resolutive therapy is available [2]. It is a severe autosomal recessive connective tissue disorder linked to mutations in the prolidase gene (*PEPD*,19cen-q13.11), which encodes for prolidase (peptidase D, EC 3.4.13.9), the only human enzyme catalyzing the hydrolysis of dipeptides containing proline or hydroxyproline residues at their C-terminal end. In humans, prolidase is a cytosolic Mn(II)-dependent homodimer of 123 kDa (493×2 amino acids), each subunit hosting a dinuclear Mn(II)-Mn(II) center [3]. A detailed description of the structural environment around the two active sites is available, whereby both enzyme subunits contribute conserved residues as ligands to the Mn(II) cofactor [4]. A partial Mn/Zn substitution has been previously reported [4].

Prolidase is widely distributed among organs and tissues, being involved in several functions, such as protein catabolism, especially collagen turnover, and regulation of collagen biosynthesis [5]. It is also involved in matrix remodeling and cell growth and, through regulation of growth and transcription factors, it plays roles in physiological and pathological processes, such as wound healing, inflammation, angiogenesis, proliferation and carcinogenesis [6], [7]. Prolidase is thought to participate in the regulation of nitric oxide biosynthesis; thus, the strong connection between prolidase and the pathways regulated by NO expands its roles in many biological processes [8]. PD patients show a reduced or absent prolidase activity in erythrocytes, leukocytes and cultured fibroblasts, with ensuing accumulation of undigested dipeptides in urine [9]. Nowadays twenty-four mutant alleles have been reported, in homozygosis or compound heterozygosis, as causative of the disease, but the bases of the clinical outcome and the genotype / phenotype relationship are still unclear [2]. The phenotypic spectrum of PD patients is broad: the clinical outcomes include dermatological manifestations with chronic, slowly healing ulcerations, recurrent infections of the respiratory tract, facial dysmorphism and different levels of mental retardation [9]. Patients die by infections as a result of severe ulcers.

During the past two decades, different approaches have been introduced for the treatment of loss of function diseases. Among them, enzyme replacement therapy (ERT) represented a major advancement that was successfully exploited in the treatment of some of these disorders [10]. However, ERT has limitations such as insufficient bio-distribution of recombinant enzymes and high costs. Emerging strategies for the treatment of this class of pathologies focus on molecular, pharmacological and chemical chaperone therapies, based on the administration of chaperone species that assist folding of mutated enzymes and improve their stability and/or correct localization [11]. After proof-of-concept studies, the chaperone therapy is now being translated into clinical applications for Fabry [12], Gaucher [13] and Pompe diseases [14]. Furthermore, a chemical chaperone has been recently demonstrated to stabilize cystic fibrosis transmembrane conductance regulator protein [15], [16], [17], and the use of monoclonal antibodies with chaperon-like activity has been proposed as treatment for Alzheimer disease and related foldopathies [18]. In the present communication we characterized the molecular bases of prolidase activity loss in three PD patients carrying in homozygosis mutations differentially located within the enzyme, namely 231delY, E412K or G448R. In particular residue Y231 falls in a solvent accessible area, within an α-helix at the dimer interface; E412 is one of the metal ligands in the active site and is poorly solvent accessible; and G448 is located in an extended β-strand, in a solvent inaccessible region close to the metal binding site. Furthermore we identified chaperone-assisted protein fold recovery as a promising approach for the treatment of prolidase deficiency.

## Materials and Methods

### Ethics Statement

All the experiments on human samples were approved by the Ethics Committee of the University of Pavia, Department of Internal Medicine and Medical Therapy on 02/08/2005,prot 22/CE, nˆ1/2005. The Ethics Committee local members were: Prof. E. Ascari, Prof. R. Fogari, Prof. G. Frigo, Prof G.R. Corazza, Prof. E. Perucca, Prof. S.B. Solerte and Dr F. Crema; the external Ethics Committee members were: Dr G. Buniva, Dr. G. Criscuoli, Prof P. Danesino, Mr G. Caronni, Mr A. Montanari, Prof. A. Marinoni, Prof. L. Vergine, Mrs A.M. Grugnetti.

### Preparation of Cultured Skin Fibroblasts

Dermal fibroblasts, from patients affected by prolidase amino acid substitutions 231delY [19], G448R [20] and E412K [20], and controls (n = 2), were established from skin punch biopsies after written informed consent. Cells were plated at 4×10^4^ cells/cm^2^ in T75 Flask in Dulbecco’s modified Eagle’s medium (Sigma Aldrich), supplemented with 10% fetal bovine serum (FBS, Euroclone), 50 U/ml penicillin (Euroclone), 50 µg/ml streptomycin (Euroclone) at 37°C in a 5% CO_2_ incubator. At confluence, fibroblasts were lysed by freezing and thawing in 1 ml of 50 mM Tris-HCl pH 7.8 and stored at −20°C. Proteins quantitation was determined by the RC DC Protein Assay (Bio-Rad).

### Human Recombinant Prolidase (hRecProl) Forms Expression and Purification

Mutant human recombinant prolidase forms (hRecPro-231delY, hRecProl-E412K and hRecProl-G448R) were obtained in prokaryotic host (*E. coli*) as previously described for the wild type protein (hRecProl-WT) [21]. Several protein preparations were needed to obtain a sufficient amount of hRecProl-G448R, due to a marked precipitation trend of this mutant. The purified proteins were extensively dialyzed against 10 mM Tris-HCl pH 7.8, 0.57 mM dithiothreitol (DTT) and 300 mM NaCl, aliquoted and stored at −80°C.

### Prolidase Activity Assay

Prolidase activity was determined at 50°C following an incubation step performed at the same temperature, as described in Besio *et al.,* at concentrations fully compatible with maintenance of the dimeric structure of the proteins [22]. The activity of the human recombinant enzyme was expressed as µmol of proline released per min per mg of protein; the activity measured in fibroblasts lysates was expressed as µmol of proline released per min per mg of total proteins. All measurements were performed in triplicate using a Jasco V-550 UV/VIS spectrophotometer. Proline in 5 mM HCl was used for quantitation.

### Kinetic Analysis and Protein Dependence on Manganese Ions

The peptide bond cleavage rate was determined incubating the recombinant enzymes with different concentrations of the Gly-Pro or Phe-Pro substrates from 0 to 0.1 M, as previously described [22]. The reaction was stopped after 10 and 30 min. The first 10 min allowed the reaction mixture to reach equal temperature and homogeneity. The amount of proline released in 20 min was calculated as difference between the proline released at 30 and 10 min, respectively. The reaction rate was calculated as the ratio between the amount of proline released and the time of reaction, normalized to the amount of protein. The kinetic parameters V_max_ (µmol Pro min^−1^ mg^−1^), k_cat_ (s^−1^), K_M_ (mM) and k_cat_/K_M_ (M^−1^ s^−1^) were determined with the Enzyme Kinetic Module 1.1 (Sigma Plot).

The binding constant for the Mn(II) cofactor was determined from the rate dependence of prolidase activity on MnCl_2_ concentration, at saturating levels of the substrate Gly-Pro, as previously reported for the wild type enzyme [22].

### Metal Content Analysis

The recombinant protein samples (0.85 µM–1.8 µM), after 48 h of extensive dialysis against 50 mM Tris-HCl pH 7.8, 300 mM NaCl, 10 mM EDTA Chelex-100-treated at 4°C, were analyzed by ICP-MS (Inductively Coupled Plasma Mass Spectrometry). The measurements were performed on three protein preparations for each recombinant form on a Perkin Elmer Mod ELAN DRC-e instrument, following the standard procedures suggested by the manufacturer.

### Thermal Stability Analysis

Wild type and mutant proteins, as obtained from the purification, or after a 20 min incubation with 1 mM MnCl_2_ and 4 mM β-mercaptoethanol, were mixed with the fluorescent dye SYPRO Orange (Sigma Aldrich) in a Thermo-Fast 96-well PCR plate (VWR International), resulting in a final protein concentrations of 5 µM (final volume 20 µl). The plate was heated at a rate of 1°C/min, from 25 to 95°C, and fluorescence was measured in 1°C increments. The reactions were performed using the real time PCR Mx3000P apparatus (Stratagene). The fluorescence signal was filtered through custom interference excitation (492 nm) and emission (568 nm) filters. The primary data (relative fluorescence intensity *versus* temperature) were fitted to standard equations describing protein thermal stability, as previously described [23].

### Dynamic Light Scattering

Dynamic Light Scattering (DLS) measurements were performed at 20°C in a DynaPro instrument (ProteinSolutions, Charlottesville, USA) after centrifuging all the protein samples at 20°C for 10 minutes at 16000×g. For each protein sample two independent experiments were performed, collecting 20 acquisitions every 30 seconds. Data were recorded with the software Dynamic V5 (DYNAMICS version 5.26.60 © Protein Solutions Inc.) which allowed the calculation of the molecular weights from the hydrodynamic radiuses experimentally observed.

### Analysis of the Dimerization Process

Based on the observation that prolidase is active only in the dimeric state, the recombinant prolidase activity was measured at substrate saturation (100 mM Gly-Pro), as described in Prolidase activity assay, at various protein concentrations ranging from 0.1 nM to 80 nM. The ratio between prolidase activity and protein concentration was plotted against protein concentration.

### Circular Dichroism (CD)

Far-UV (190–260 nm) CD measurements were performed at 20°C in 0.1 cm pathlength quartz cuvette. CD spectra were recorded on a Jasco J-720 spectropolarimeter at a scan rate of 50 nm/min with a 1 nm spectral band width, and collecting points every 0.2 nm. All the spectroscopic measurements were performed in 50 mM Tris-HCl pH 7.8. Measurements were performed on protein preparations concentrated at 0.2 mg/ml. Spectra were recorded also in the presence of 0.57 mM DTT, and after a 20 min incubation at 50°C with 1 mM MnCl_2_ and 0.57 mM DTT. Twenty scans were averaged for each spectrum. The results were expressed as the mean residue ellipticity. The secondary structure content was estimated from the CD spectra using the deconvolution algorithms CONTIN [24], CDSSTR [25] and SELCON3 [26] with the data set 4 at the DICHROWEB server [27], [28].

### Fluorescence Spectroscopy

Fluorescence spectra were measured with a Jasco FP-6500 spectrofluorimeter, using a 0.2 cm quartz cell at room temperature. Measurements were performed on 0.2 mg/ml protein preparation. Emission spectra were recorded in the range 290–400 nm at the excitation wavelengths 270, 280 and 295 nm, with a scanning rate of 500 nm/min.

### Limited Proteolysis

Recombinant proteins (10 µg) were digested in a reaction volume of 100 µl with 250 µg/ml α-chymotrypsin (Cooper Biomedical) in 50 mM Tris-HCl, pH 7.8, at 37°C; with 0.064 U/ml papain (Sigma Aldrich) in 0.1 M sodium acetate, pH 5.6, 5 mM cysteine, 5 mM EDTA at 4°C; with 120 µg/ml trypsin (Sigma Aldrich) in 1 mM HCl at 37°C. After 0, 5, 30, 60 min the reactions were stopped by adding Laemmli sample buffer (60 mM Tris-Cl, pH 6.8, 2% SDS, 10% glycerol, 0.1 M DTT, 0.01% bromophenol blue) and immediately heated at 90°C. The proteolytic patterns were analyzed by 10% SDS-PAGE under reducing conditions. Coomassie blue was used for staining.

### *In silico* Analysis

To analyze the prolidase dimerization interface the software PISA [29] was used, based on the crystal structure of human prolidase deposited in the Protein Data Bank (ID 2OKN) [30]. The online software http://www.predictprotein.org/was used for secondary structure predictions. All the structure images were drawn with Pymol [31].

### Heat Shock Proteins Stimulation

Dermal fibroblasts (6.6×10^3^ cells/cm^2^) were plated in 35 mm Petri dishes to 90% confluence, as described previously. DMEM without L-glutamine was heated until it equilibrated to 48°C. The cells were rinsed with PBS to prevent cell damage caused by the degradation of L-glutamine at high temperatures. PBS was removed and flasks were filled with 10 ml of the pre warmed medium and incubated at 48°C in a 5% CO_2_ incubator from 0 to 20 min. Subsequent to the thermal stimulation, the medium was immediately removed and cells were washed with PBS. Culture medium containing 10% FBS was then replenished and the dishes were returned to 37°C to allow heat shock proteins expression [32]. 16 h post heating, the time corresponding to maximum Hsps expression, as previously reported [32], [33], cells were collected and lysed. Total proteins were extracted with 10 mM Tris-HCl pH 7.6, 5 mM EDTA pH 8.0, 140 mM NaCl, 0.5% Nonidet-P40 added with protease inhibitors (130 mM benzamidine, 2 mM NEM, 5 mM EDTA, 1 mM PMSF) and 1.6 mM Na_3_VO_4_. The fibroblasts lysates were used to evaluate both prolidase expression and activity, and Hsp70/90 protein level. Experiments were performed at least in triplicate.

### Prolidase and Hsp70/90 Expression

Proteins from fibroblast lysates (20 µg) were separated on 10% SDS-PAGE under reducing conditions and were electro-transferred to a PVDF membrane (Hybond-P, Amersham Biosciences) at 100 V for 2 h. After washing with TBS-T solution (20 mM Tris, 500 mM NaCl, pH 7.5, 0.1% Tween) membranes were incubated for 1 hour at room temperature with 5% dried milk in the same buffer. Primary antibodies against human prolidase (provided by Dr. J.M. Phang, Laboratory of Comparative Carcinogenesis, NCI-Frederick MD, USA) and Hsp70 (Abcam) were diluted 1∶3,000 and 1∶2,500, respectively, in TBS-T containing 5% milk, while Hsp90 (Abcam) was diluted 1∶500 in TBS-T containing 3% BSA. The primary antibody incubation was performed overnight at 4°C whereas the secondary antibody conjugated with horseradish peroxidase (ECL anti-mouse Peroxidase Labelled, Amersham, GE Healthcare; donkey anti-rabbit IgG-HRP, Santa Cruz) was incubated at room temperature for 1 h. The signal was detected with ECL Western Blotting Detection Reagents (Amersham, GE Healthcare). Films were acquired by VersaDoc 3000 (BioRad), and band intensity evaluated by QuantityOne software (BioRad). α-tubulin was used for protein load normalization.

### Statistical Analyses

The values of the kinetic parameters were expressed as mean ± SD of at least three different measurements obtained from three independent protein preparations.

Statistical comparisons were performed by t test analysis. A p-value less than 0.05 was considered statistically significant (two-side). The analyses were performed using SigmaPlot Statistics 11.0.

## Results

**Figure 1:**
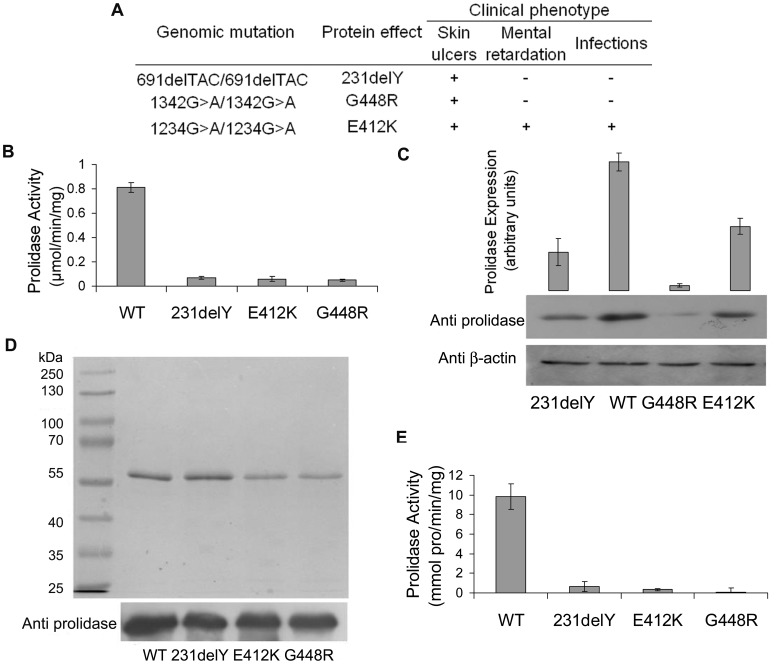
Human prolidase activity and protein expression. (A) Mutations and clinical phenotype of the PD patients used for the study. (B) Prolidase activity levels, and (C) prolidase expression levels in control (WT) and PD patients fibroblasts lysates (231delY, E412K, G448R). Densitometric analysis of Western blots (C, top) shown in the lower part of the panel. (D) SDS-PAGE (top) and Western blotting (bottom) of purified recombinant WT and mutant forms. (E) Prolidase activity of the recombinant proteins.

In order to better clarify PD etiology, we selected three naturally occurring mutations, previously characterized in homozygous conditions in our patients cohort, 691delTAC (231delY), 1234G>A (E412K) and 1342G>A (G448R) [19], [20] (**Figure 1A**); we then produced and purified the respective recombinant enzymes (**Figure 1D**) for structural and kinetic characterization. All the selected mutations were located in the prolidase domain belonging to the M2 Metal Peptidase homology family.

### Prolidase Activity and Expression in PD Patients’ Fibroblasts

Prolidase activity in PD patients’ fibroblasts, carrying in homozygosis the selected amino acid substitutions, was found to be strongly reduced, with maximum values reaching only about 8.5% of the controls (**Figure 1B**). Protein content analysis showed also reduced prolidase expression in all samples (**Figure 1C**), although enzyme activity and expression were not correlated. In the E412K lysate the presence of 50% prolidase provided 7.4% of its normal activity; in 231delY a 30% enzyme abundance yielded 8.5% activity, and in G448R a 4% prolidase content corresponded to 6.1% of activity (**Figure 1** **B,C**). These data strongly supported the conclusion that the molecular bases of the functional defects required thorough elucidation through fundamental biochemical investigations, such as prolidase kinetics and structural considerations.

### Mutant hRecProl Forms Generation and Kinetic Analysis

All the pathological variants were expressed in *E.coli* (hRecProl-231delY, hRecProl-E412K and hRecProl-G448R) and purified following the protocol previously optimized for the wild type enzyme [21]. The recombinant proteins revealed a single band of 57 kDa in SDS-PAGE, indicating an overall purity >95% (**Figure 1D**), and were properly recognized by a specific antibody against human prolidase (**Figure 1D**). Their activity was about 2–7% of the corresponding wild type form, in agreement with the prolidase activity values detected in PD patients’ cells (**Figure 1E**). The activity of the hRecProl-G448R mutant was difficult to detect due to protein instability, which strongly reduced protein recovery, reminiscent of what had been observed for the endogenous mutant form.

**Figure 2:**
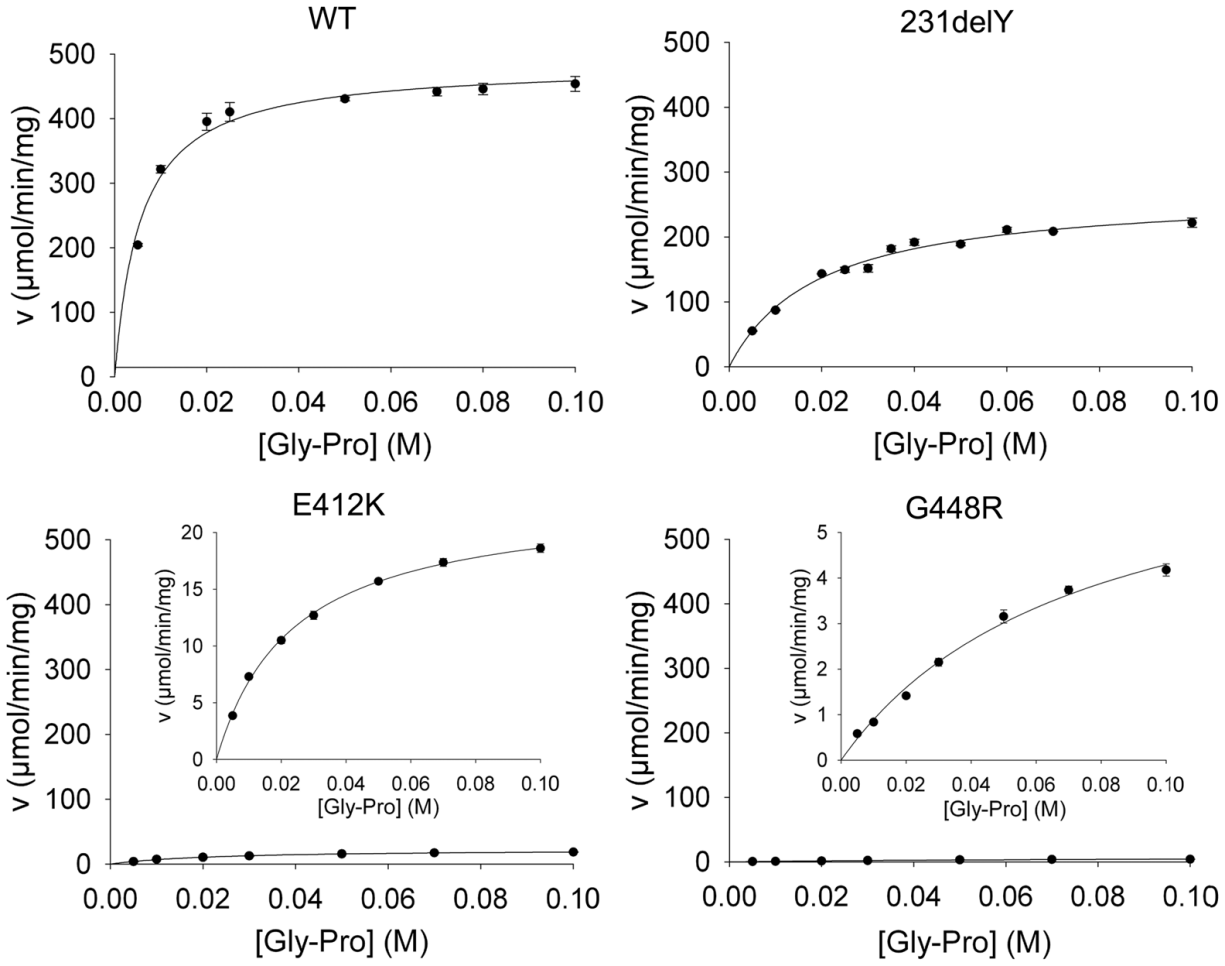
Kinetic analyses of hRecProl-WT and its pathological variants. Michaelis-Menten plots obtained using Gly-Pro as substrate. The graphs of hRecProl-E412 and hRecProl-G448 were magnified in the insets for clarity.

Kinetic analyses were performed for all variants, at a fixed concentration of the Mn(II) protein cofactor in the assay solutions, using two different prolidase substrates: Gly-Pro, the natural preferred substrate, and Phe-Pro, which allows to investigate the accessibility of the mutant active site to a bulkier substrate. All the pathological enzymes exhibited Michaelis-Menten kinetic (**Figure 2** **,** **Table 1**), but showed very low catalytic efficiency with respect to the wild type prolidase. Relative to the Gly-Pro substrate, the hRecProl-231delY variant exhibited a higher K_M_ and a reduced V_max_ value (by 50%), suggesting a perturbation of the active site structure in this mutant. hRecProl-E412K showed a reduced V_max_ (98% decrease) and a low affinity for the Gly-Pro substrate (86% decrease). Interestingly, for both the hRecProl-231delY and hRecProl-E412K variants the K_M_ value measured for Phe-Pro was comparable to that of the wild type protein (**Table 1**). hRecProl-G448R revealed a substantial K_M_ increase for both substrates; the *k*_cat_ values obtained for Gly-Pro and Phe-Pro (65 and 8.8 times lower than WT prolidase, respectively) were indicative of a substantial perturbation of the active site structure.

**Table 1:** Kinetic properties of hRecProl-WT and its pathological variants.

GLY-PRO	WT	231delY	E412K	G448R
V_max_ (U/mg)	489±12	261±13	29±2	6.5±0.3
K_m_ (mM)	5.41±0.01	17.87±0.01	38.05±0.01	59.5±0.1
K_cat_ (s^−1^)	680±10	429±20	52±4	10.3±1.2
K_cat_/K_m_ × 10^−3^ (M^−1^s^−1^)	126±19	24±2.6	1.4±0.13	1.7±0.1
**PHE-PRO**	**WT**	**231delY**	**E412K**	**G448R**
V_max_ (U/mg)	58±3	22.3±1.5	21±2	8±1
K_m_ (mM)	4.3±1.2	6.5±1.9	5.7±2.6	48.2±0.9
K_cat_ (s^−1^)	100±12	41.6±8.2	32.9±1.2	11.4±1.4
K_cat_/K_m_ × 10^−3^ (M^−1^s^−1^)	23.3±5.1	6.5±1.4	5.82±0.19	0.21±0.03

### Effects of the Mutations on the Affinity for the Mn(II) Cofactor

In the hRecProl-231delY one Mn(II) was tightly bound, as observed for the WT enzyme (**Table 2**), while the affinity constant for the weakly bound Mn(II) ions was nine times lower (6.4±1.4 mM^−1^ versus 54±10 mM^−1^ detected in hRecProl-WT), indicating that the mutation perturbed the active site. Cofactor binding was strongly affected also in the mutant hRecProl-G448R, for which no metals were detected after dialysis in the presence of 10 mM EDTA (**Table 2**). In fact, contrary to what occurred for the WT enzyme, the chelating agent removed also the more tightly bound Mn(II), suggesting either a decreased affinity for this ion, or an increased solvent accessibility. In the absence of EDTA, the K_B_ for the weakly bound ion (39±15 mM^−1^) was instead comparable with that of the wild type protein. Conversely, the hRecProl-E412K variant displayed two tightly bound Mn(II) ions per dimer (**Table 2**), and a slightly increased metal binding affinity (103±42 mM^−1^), in keeping with minor effects of the E412K mutation on the cofactor coordination shell.

**Table 2:** Prolidase metal content.

	Protein (nmol)	Mn(II) (nmol)	Zn(II) (nmol)	Mol dimer:Mol Mn(II)	Mol dimer: Mol Zn(II)
**WT**	32	28±3	90±9	∼1∶1	∼1∶3
**E412K**	17	44±2	33±1	∼1∶2.5	∼1∶2
**231delY**	24	17±9	44±9	∼1∶0.7	∼1∶1.8
**G448R**	36	3±1	33±13	∼1∶0	∼1∶1

Mn(II) and Zn(II) content was analyzed by ICP-MS after 48 h dialysis against metal free buffer based on their presence in the enzyme active site as previously demonstrated [4].

### Thermal Stability

**Figure 3:**
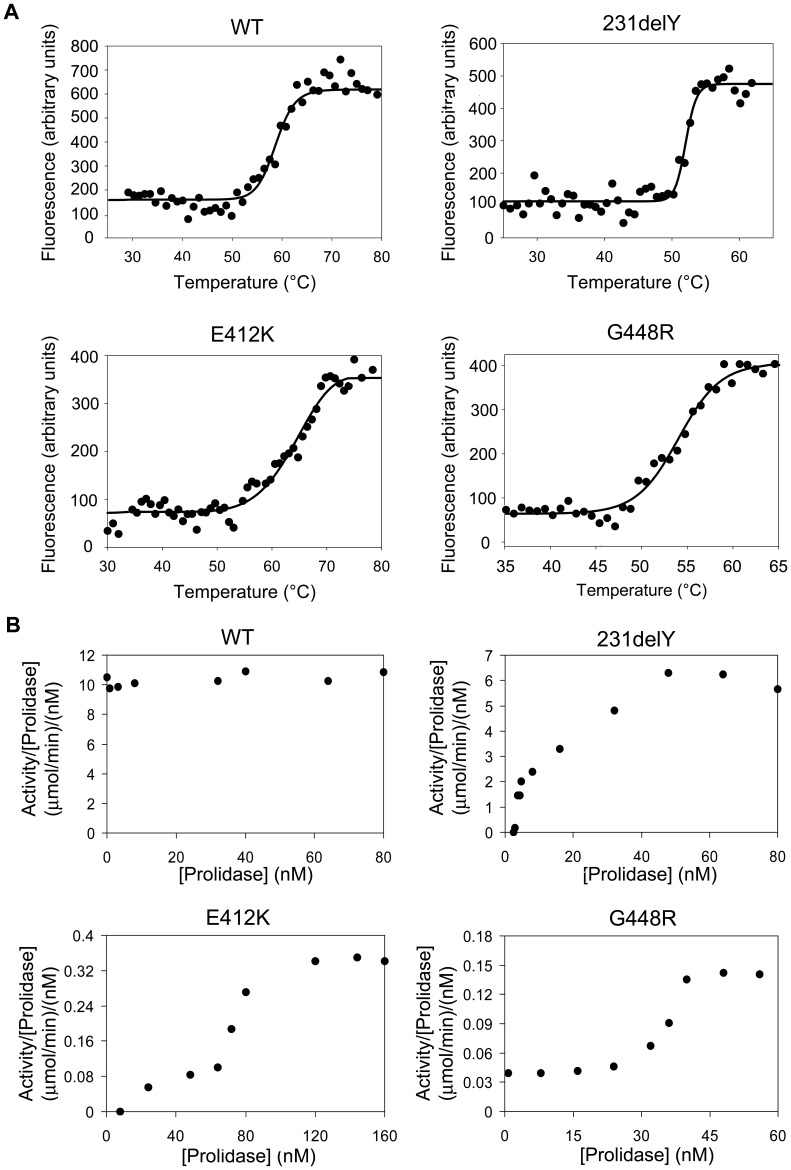
Prolidase thermal denaturation and dimerization follow up. (A) Protein melting temperatures in the presence of the cofactor as revealed by Thermofluor Technology. The solvatochromic dye SYPRO orange was used as an indicator of protein unfolding (fluorescence excitation λ = 492 nm; fluorescence emission λ = 568 nm). (B) The ratio between prolidase activity and protein concentration was plotted against protein concentration.

The WT and mutant prolidase forms, following incubation with Mn(II), were heat-denatured in a one-state process. hRecProl-231delY and hRecProl-G448R showed a reduced T_M_ (55°C and 54°C respectively) compared to WT prolidase (60°C), while hRecProl-E412K had a T_M_ of 64°C (**Figure 3A**). The protein stability in absence of the cofactor was also evaluated, revealing reduced T_M_ values for all the mutants (hRecProl-231delY T_M_ = 55°C, hRecProl-E412K T_M_ = 51°C, hRecProl-G448R T_M_ = 49°C) with respect to hRecProl-WT (T_M_ = 57°C), which was also reduced of 3°C (**Figure S1**). hRecProl-G448R showed the lowest T_M_, in agreement with the low recombinant protein purification yield, and with the low protein levels detected in PD patients fibroblasts.

### Prolidase Dimerization in the Mutant Species

DLS data on Mn(II)-activated WT and mutant prolidase forms (at concentration of ca. 5 µM) showed that the protein samples displayed hydrodynamic radiuses indicative of a dimeric prolidase (hRecProl-WT, 5.7±0.1 nm; hRecProl-231delY, 5.0±0.2 nm; hRecProl-E412K, 5.25±0.05 nm; hRecProl-G448R, 5.0±0.3 nm, Table S1) with a polydispersion of less than 30%.

In order to assess the enzyme quaternary assembly at lower concentrations, where the DLS analysis fails, the dependence of prolidase activity on protein concentration was monitored. For WT prolidase the specific activity did not change indicating that, the enzyme was present in the dimeric state even at low nanomolar concentrations (Figure 3B). On the contrary, the activity of all the mutant enzymes was markedly concentration dependent (Figure 3B).

### Structural Analysis of Prolidase Variants

**Figure 4:**
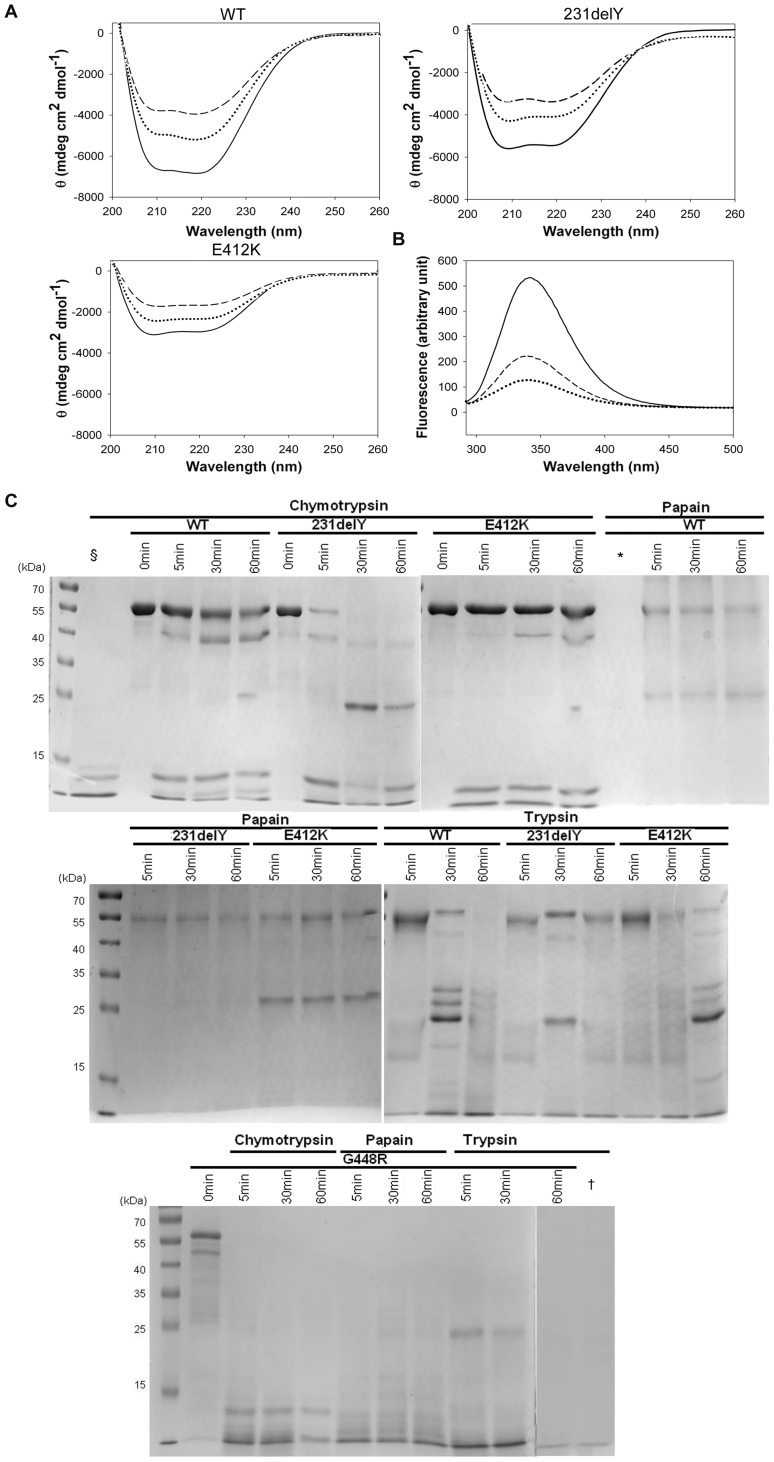
Prolidase spectroscopy analysis. (A) CD spectra of hRecProl-WT, hRecProl-231delY and hRecProl-E412K in the far-UV spectrum region (__). Spectra were recorded also in the presence of 0.75 mM DTT (·····) and of both 0.75 mM DTT and 1 mM MnCl_2_ (­­­). The reduction in signal intensity was due to a slight loss of the protein in solution. (B) Fluorecence spectra of hRecProl-WT (__) and its variants hRecProl231delY (­­­) and hRecProl-E412K (····). The tryptophan excitation wavelength was set at 295 nm, the monitoring emission from 305 to 400 nm. The other spectra recorded at the wavelengths specific for phenylalanine and tyrosine were similar and thus not reported. (C) Limited proteolysis analyses of mutant prolidase. Digestions were performed with α-chymotrypsin, papain and trypsin. The fragmentation patterns were analyzed by SDS-PAGE under reducing conditions and stained with Comassie blue. In lines §, *and ^†^only chymotrypsin, papain and trypsin were loaded, respectively.

The secondary structures of wild type and mutant prolidases were analyzed by circular dichroism (CD). Far-UV CD spectra of all proteins showed two minima, at 205–210 and at 215–220 nm respectively (**Figure 4A**). Deconvolution of the spectra using three independent algorithms indicated for the WT enzyme 23% α-helix, 28% β-sheet and 23% coil relative contents, while the spectra of hRecProl-E412K and hRecProl-231delY showed an increased contribution of the random coil signal, reflected by a reduction of the peak at 220 nm (**Table 3**), in keeping with an effect of the mutated residues on proper enzyme folding.

**Table 3:** Secondary structure composition as estimated by the CD spectra of the recombinant prolidase variants.

	WT	231delY	E412K
**α-Helix**	0.23±0.01	0.16±0.05	0.18±0.10
**β-Sheet**	0.28±0.07	0.28±0.04	0.33±0.07
**Turns**	0.23±0.03	0.24±0.01	0.19±0.12
**Random coil**	0.26±0.03	0.32±0.01	0.30±0.11

CD spectra were also recorded in the presence of the reducing agent DTT, and after a 20 min incubation at 50°C in the presence of Mn(II) and DTT [22]. Both incubation conditions did not affect the CD spectra, indicating that the metal cofactor was not responsible for secondary structure variations. On the other hand, the fine structural properties of the mutated proteins, as evaluated by fluorescence spectroscopy, indicated the presence of some conformational changes, since aromatic residues in hRecProl-E412K and hRecProl-231delY appeared more exposed to solvent than in the WT enzyme (**Figure 4B**). For the mutant hRecProl-G448R it was not possible to perform CD and fluorescence spectroscopy experiments due to the low protein amount obtained from the purification.

To investigate the hRecProl-G448R stability, and to further support the conformational changes revealed in hRecProl-231delY and hRecProl-E412K, distinct limited proteolysis experiments were performed using α-chymotrypsin, papain and trypsin. Different proteolytic patterns were identified in all mutated forms, indicating their different sensitivity to proteolytic cleavage (**Figure 4C**). During chymotrypsin digestion, full length hRecProl-231delY was strongly degraded after just 5 minutes, with the appearance, after 30 min, of a 25 kDa fragment undetected in the WT prolidase. After trypsin digestion, hRecProl-231delY also revealed faster proteolysis, characterized by the absence at 30 min of the ∼27 and 32 kDa bands present in the WT protein. Interestingly, hRecProl-E412K was more resistant than the WT enzyme to both chymotrypsin and trypsin proteolysis. Although enough hRecProl-G448R mutant was available for limited proteolysis analysis, this variant proved very unstable and was completely degraded by all the tested enzymes after just 5 min incubation (**Figure 4C**).

### *In silico* Analysis of Mutants: Structural Prediction

**Figure 5:**
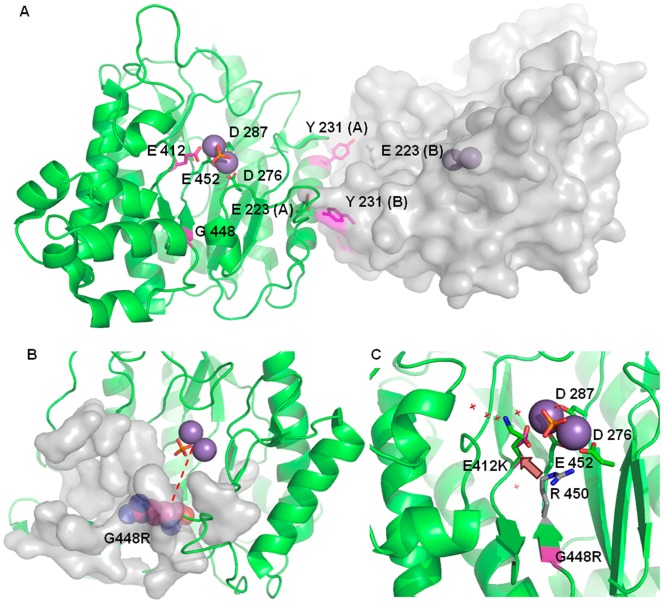
Mutant prolidase forms *in silico* modeling. (A) Human prolidase dimer: the two prolidase interfacing monomers are reported as a green cartoon (molecule A) and as a grey surface (molecule B), with Mn(II) shown as dark grey spheres. The magenta color was chosen to identify mutated residues. On molecule A, Mn(II) coordinating residues (D276, D287, E412 and E452) and the phosphate group (orange) are highlighted as sticks. Y231 residues from molecules A and B are located on the C-terminal portion of two α-helices arranged in a symmetric and anti-parallel manner, allowing their interaction with E223 of the interfacing protomer. (B) Destabilization of prolidase core structural elements upon G448R mutation. R448 substituenting residue is reported in spheres to highlight its steric hindrance. The protein portion that buries the mutated residue is shown as a grey surface. A red dashed line indicates the distance between the average position of Mn(II) ions and the C_α_ of the substituted residue. (C) E412K substitution does not impair ions binding. Solvent molecules occupy a small cavity near the active site, and are represented with red crosses. This small cavity can host the side chain of the mutated K412 (reported in sticks with green carbon atoms and the nitrogen atom in blue) which substitutes E412 (magenta carbon and red oxygen atoms). The putative location of the K412 side chain here proposed may be set by the electrostatic repulsion exerted by residue R450 (shown in sticks with grey carbon atoms).

The dimeric assembly of human prolidase (molecules A and B), as reported by the enzyme crystal structure (PDB ID 2OKN), is stabilized by different structural motifs; these include α-helices and coil regions, producing a total interface area of 2861 Å^2^, characterized by a calculated solvation free energy (Δ^i^G) of −25.7 kcal/mol. Residue Y231 is located in one of the two α-helices involved in association, and represents ∼3.1% of the total interface, with a buried surface area of 88 Å^2^. The Y231 hydroxyl group, in both protein subunits (A and B), is hydrogen bonded to the side chain of E223 (Y231 A/B *{OH}* – E223 B/A *{OE1}* = 2.52 Å) from the facing protein subunit, contributing to the stabilization of the associated dimer **(** **Figure 5A**).

The calculated residue solvation free energy (Δ^i^G) is slightly positive (0.8 kcal/mol), indicating that Y231 prefers hydrophobic environment and its location in a buried area is energetically favored.

Since modeling of the Y231 deletion on the WT prolidase structure may lead to inaccurate predictions, on a purely indicative way we considered only the effects of Y231 substitution on subunit association. In fact, the Y231A substitution in both protein monomers produced a decrease in the total interface area (A_Y231A_ = 2784 Å^2^) and an increase of the solvation energy (Δ^i^G = −24,1 kcal/mol) indicative of a slightly less favorable stabilization process relative to the wild type protein. The effects of a full amino acid deletion at site 231 were expected to be more dramatic.

Residue G448 is buried in a protein region, not accessible to solvent. The residue lies at about 14.5 Å from the active site and is not directly involved in Mn(II) binding. G448 belongs to a β-strand anti-parallel to a short neighboring strand composed of G414, I415, Y416, F417 (**Figure 5B**). The G448R substitution forces the insertion of a bulky arginine side chain (**Figure 5B**), which is not compatible with pairing of the two anti-parallel β-strands and with the correct assembly of the β-sheet. Furthermore, the G448R mutation falls only four amino acids before residue E452, that coordinates one of the Mn(II) cofactor ions; therefore, a perturbation of the ligand coordination sphere upon G448R substitution cannot be excluded. Such an observation is in keeping with the absence of metal ions experimentally detected for this mutant by ICP-MS.

In WT prolidase the Mn(II) ions are coordinated by the negatively charged amino acids D276, D287, E412, E452, and by a phosphate group. Thus the E412K mutation decreases by two units the negative charge in the coordination sphere; nevertheless, our *in vitro* data showed that the mutation does not affect the binding of the Mn(II) cofactor. Modeling considerations suggested that K412 side chain, due to electrostatic repulsion by R450, can relocate into a solvent area adjacent to the active site, and such accommodation would not impair the Mn(II) coordination surroundings (**Figure 5C**). Since the three-dimensional structure of the enzyme in the presence of a substrate analogue is not available, any structural prediction on K412-dependent impairment of substrate binding would be largely hypothetic.

### Mutant Proteins Stabilization through Heat Shock Proteins Induction

**Figure 6:**
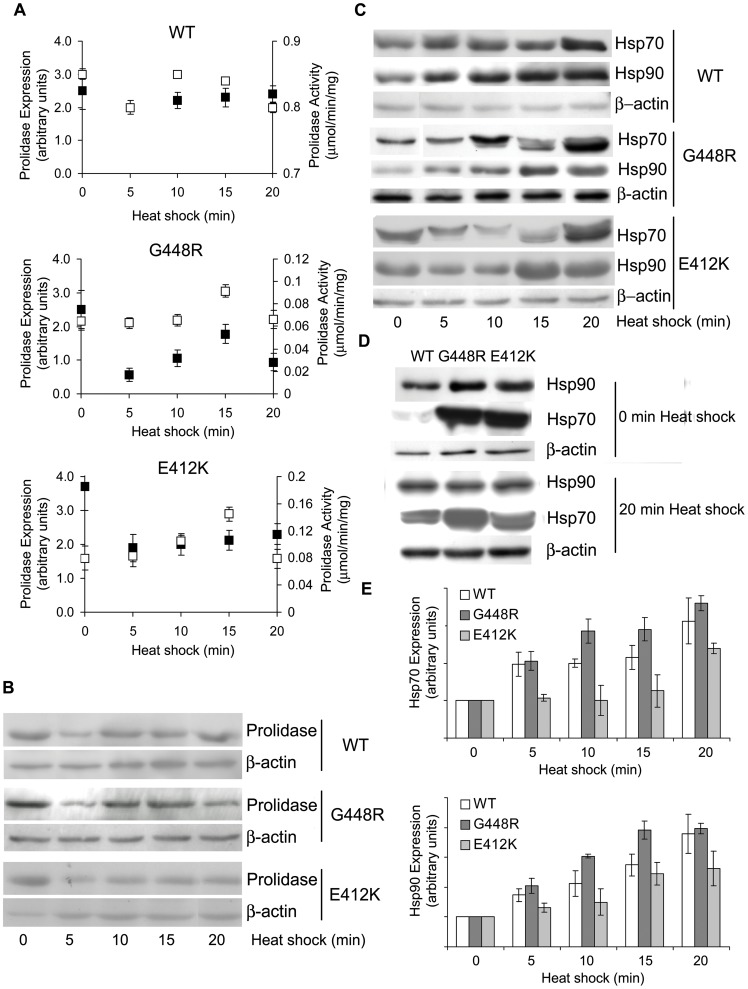
Mutant prolidases stabilization though heat shock proteins induction. (A) Prolidase activity (white square) and protein expression level (black square) of WT, G448R, E412K fibroblasts lysed 16 h after the heat shock (at 48°C).*, p<.05. (B) Representative western blot performed to evaluate the prolidase expression in WT, G448R, E412K fibroblasts 16 h after the heat shock. (C) Hsp70/90 expression in WT, G448R, E412K fibroblasts 16 h after the heat shock. (D) Comparison of Hsp70/90 expression in WT, G448R and E412K at time zero, and after 20 min heat shock. The same exposure time was used in order to emphasize the different expression levels. (E) Densitometric analysis of the Hsp70/90 expression as presented in (C). The proteins expression was quantified by using Quantity One software.

Our findings led us to consider enzyme stabilization as a means for rescuing prolidase activity *in vitro*, with the aim to evaluate it *in vivo*. To test the feasibility of this approach, we chose to stimulate expression of the endogenous heat shock proteins (Hsps). Hsps are molecular chaperones that in the presence of adverse environmental conditions, assist refolding of misfolded proteins and facilitate the synthesis of new proteins to enhance cell survival [34]. *In vitro* Hsps stimulation was performed by means of heat increase: fibroblasts from patients homozygous for 231delY, G448R and E412K, and from controls, were heated at 48°C, for periods ranging from 0 to 20 minutes and, sixteen hours post-heating, prolidase activity and protein expression level were evaluated at selected time points. Although the prolidase activity was expectedly low in all the mutants, with respect to the controls, and although a partial loss of the protein was caused by the heating procedure as evidenced by Western blot analysis, following 15 min of heat shock in the G448R lysate the prolidase activity was increased by 22% relative to the same cells not stimulated. An even better result was obtained for the E412K mutant that showed a prolidase activity increase of 40% (**Figure 6A, B**). No increase in activity was instead detected in control lysates (**Figure 6A, B**).

Since the two major known heat inducible cytosolic chaperones involved in protein folding are Hsp70 and Hsp90 [35], an analysis of their protein expression levels was performed. A significant increase of both proteins was detected after stimulation in all the analyzed cell lines, with the highest level following 20 min heat shock (**Figure 6C, E**). Both constitutive and inducible forms of Hsp70 were detectable. The latter one started to be synthesized after 10 min heating and reached its maximum after 20 min (**Figure 6C, E**). Although the Hsps expression increased by prolonging the heat shock up to 20 min, prolidase activity reached its maximum following 15 min and then decreased (**Figure 6A, B**). Also in control cells the incubation at 48°C for 20 min produced a slight reduction of prolidase activity, likely indicating that, under such stressful conditions, even if the chaperone levels increase, irreversible cellular damages occurred (**Figure 6A, B**) [36]. As expected, in mutant cells, Hsp70 was expressed at steady state at higher concentration relative to the control (**Figure 6D**). Unfortunately, an increase in activity following heat stimulation was not evident for 231delY variant (data not shown), revealing that the efficacy of the Hsp-mediated refolding process may be mutation specific.

## Discussion

Twenty-four mutant alleles have been reported to date as responsible for PD, however, the identification of the causative/pathologic genetic defects in the prolidase gene does not immediately translate into an understanding of the molecular basis of the disease. How specific mutations affect the enzyme activity is still an open question in the field. Although the residues mutated in the pathologic variants are strictly conserved in prolidase from different organisms (**Figure S2**), their contributions to the hydrolytic reaction and/or to the enzyme structure have not been thoroughly analyzed yet.

For the biochemical investigation of the PD pathophysiology we selected three mutations previously characterized in our patients cohort, namely 691delTAC (231delY), 1234G>A (E412K) and 1342G>A (G448R) [19], [20]. The three variants were selected based on their occurrence frequency, and in order to map different, but highly conserved regions within the prolidase structure (**Figure S2**). The enzyme variants from PD patient fibroblasts revealed a strongly reduced prolidase activity and variable degrees of protein expression levels (**Figure 1B, C**), suggesting that observed impaired prolidase activity was only partly due to decreased protein expression, which proved to be mutation specific.

The relationship among enzyme activity, protein levels and phenotype was also puzzling. All patients showed severe ulcers, but only the patient carrying the E412K mutation was also affected by respiratory infections and mental retardation (**Figure 1A**) [19], [20].

All the pathological recombinant enzymes exhibited a Michaelis-Menten kinetic (**Figure 2**), but showed very low catalytic efficiency with respect to wild type using both Gly-Pro and Phe-Pro substrates. Since also hRecProl-WT displayed modest activity against Phe-Pro (**Table 1**) [37], the effects of the mutations appear more sizeable when the enzyme was tested against the naturally preferred Gly-Pro substrate. In hRecProl-G448R the kinetic parameters were indicative of a substantial active site structural perturbation, while the results on the hRecProl-231delY suggested that the deletion may perturb the substrate positioning/access to the catalytic site with a consequent decrease in the rate of catalysis. For the hRecProl-E412K, a higher K_M_ value indicated that, besides being a ligand of the metal [38], residue 412 may also be involved in substrate binding/access.

Since the Mn(II) ion is a key element in prolidase catalytic mechanism, the effect of the mutations on cofactor binding was analyzed. As previously described, one Mn(II) ion was found to be tightly associated with the wild type enzyme [4], while no metals were detected after dialysis in hRecProl-G448R, and in the hRecProl-231delY cofactor binding was strongly affected. Conversely, in hRecProl-E412K two Mn(II) ions were found as tightly associated, and the K_B_ was not significantly different from that of the WT protein, in keeping with minor predicted structural effects of this mutation on the Mn(II) coordination environment, and despite the 98% loss of enzymatic activity. Notably, a residue structurally homologous to E412, recognized in aminopeptidase P (APPro, EC 3.4.11.9, E383), in methionine aminopeptidase (MetAP, EC 3.4.11.1a, E204) and in prolidase from *Pyrococcus furiosus (Pf*Prol, E313), was shown to be essential for catalysis: mutation of E383 reduced porcine APPro, *E.coli* APPro and *E. coli* MetAP prolidase activities by at least two orders of magnitude [39], [40]. Similarly, the E313L mutation in *Pf*Prol produced an inactive highly misfolded protein, recalcitrant to purification [41], and in the APProE383A mutant, despite the impaired activity, both Mn(II) ions were still present in the active site [42]. Thus, our data, together with findings from the homologous enzymes, suggest that residue E412 contributes to a general structural maintenance of the active site region [43], [44]. The prolidase catalytic mechanism is still largely uncharacterized but, considering that the enzyme belongs to the “pita-bread” family, Lowther & Matthews proposed a common reaction mechanism [44] whereby the metal ion would coordinate the substrate, polarizing its carbonyl group, and the glutamate-activated bridging water molecule would provide the nucleophile hydroxyl group, setting conditions for hydrolysis of the bound peptide [45], [46], [47]. Our kinetic data on the hRecProl-E412K mutant (E383 in APPro) are in keeping with such water molecule-activator role for residue E412.

A reduced thermal stability was detected in all three mutant prolidase forms. Interestingly, a stabilizing effect of the Mn(II) incubation was detected in hRecProl-WT (Δ_TM_ = +3°C), in hRecProl-G448R (Δ_TM_ = +5°C) and in hRecProl-E412K (Δ_TM_ = +13°C), whereas no effect was detected in hRecProl-231delY. The higher stabilization of hRecProl-E412K, and the absence of stabilization in hRecProl-231delY, are in keeping with the Mn(II) cofactor binding properties observed for these mutants.

The monitoring of the ratio between prolidase activity and protein concentration plotted against protein concentration revealed that while WT prolidase was active in solution as a dimer, in the mutant enzymes the activity was concentration dependent. At very low concentrations (<4 nM), in keeping with the loss of enzymatic activity, the two subunits appeared dissociated in all variants; at slightly higher concentrations the enzymes were presumably present as a mixed population of active dimers and inactive monomers, while at higher protein concentrations (48 nM for hRecProl-231delY, 120 nM for hRecProl-E412K and 43 nM for hRecProl-G448R) recovery of the prolidase activity indicated the presence of the dimeric species (**Figure 3B**). For multimeric proteins, subunit aggregation is often a key process in enzyme activation, and the failure or the decreased association efficiency can translate into reduced activity. Although our activity tests were performed on the recombinant prolidase forms, and although the concentration of active prolidase in the cell is not known, dimerization impairment in all the mutant enzyme species helps explain their compromised activity.

CD spectra revealed a decrease in α-helix content in the hRecProl-231delY and hRecProl-E412K variants, indicating that both mutations have an impact on prolidase secondary structure (**Figure 4A**); tryptophan emission fluorescence spectra, together with proteolytic digestion experiments, confirmed that the pathological proteins host partly altered conformations (**Figure 4B, C**); local structure perturbations for all the mutants were also predicted by our *in silico* analyses. Based on structural considerations, Y231 was indicated as a key residue for stabilization of the prolidase dimer; deletion of this amino acid, coupled to loss of its bulky side chain that is involved in interface interactions, may destabilize one α-helix at the dimer association interface and affect the Δ^ι^G for assembly of the dimeric species.

*In silico* investigation suggested that in hRecProl, the preservation of the G448 residue may be crucial for the overall enzyme architecture, since G448 lies in a β-sheet buried in the prolidase core region, and because bulkier residues would not be compatible with the compactly packed surroundings. Conversely, due to lack of a three-dimensional structure of the enzyme in the presence of a substrate analogue, any structural prediction on K412-dependent impairment of substrate binding would be largely hypothetic, leaving a discussion on the molecular basis of reduced activity for this mutant purely speculative.

All the analyzed mutations introduced significant changes in the protein that turned into evident effects on the enzyme kinetics: even structural alterations outside the active site, as in the cases of G448R and 231delY, were translated to the catalytic center with impact on the enzyme activity. Thus, based on the data here reported, we considered that, as for many loss-of-function diseases, PD pathogenicity is strictly linked to occurrence of a perturbed protein fold, resulting in faster protein degradation that would trigger system failure. Ensuring accuracy in protein folding is a crucial aspect for proper maintenance of cellular functionality and indeed, in the cell, molecular chaperones recognize protein substrates in non-native states and assist their proper re-folding and assembly. We therefore chose to stimulate heat shock proteins (Hsps), a class of molecular chaperones expressed under sublethal stressful stimuli, that have previously been identified by several studies as defense components enhancing cell survival under stress conditions [32], [48], [49], [50].

As a result of heat stimulation, fibroblasts bearing either the G448R or the E412K mutations revealed a decrease in prolidase content, but a statistically significant increase in prolidase activity (relative to fibroblasts carrying the same mutation, but not heat stimulated). No beneficial effects were instead detected in heat stimulated fibroblasts bearing the 231delY mutation, suggesting that the effects achievable through Hsp-based recovery are mutation specific. The Y231 deletion, impairing prolidase dimerization, may be one of the reasons explaining the failure of Hsp stimulation approach. Interestingly, it was noted that in the absence of heat stimulation the level of the Hsp70 expression was higher in G448R and E412K mutant fibroblasts than in the controls, suggesting that cells specifically activate the Hsp endogenous response to help folding the mutant prolidase forms, or more generally to respond to the ensuing cellular stress [51]. Thus, pharmacological stimulation of the Hsp expression appears as a therapeutic approach in line with the natural response of the cell.

Altogether, our data suggest that a moderate temperature increase lead to enhance degradation of the poorly structured prolidase mutants, minimally affecting the WT form. Improved stability of the mutant enzymes was found when higher chaperone levels were present. Such stabilization translated into an increase in mutant prolidase activity and, even if the prolidase activity after Hsp stimulation remained lower than that of the WT enzyme, these findings suggest that drugs effecting the intracellular chaperone environment, some of which are already FDA approved [11], could be tested for PD treatment.

### Conclusions

Our study on three pathological mutant human prolidase forms showed that a substantial loss of enzymatic activity can be linked to enzyme instability and local structural perturbations, partly affecting the Mn(II) cofactor site; partial degradation of the mutated enzyme is an additional factor contributing to the general loss of enzyme activity in PD patients. On these bases, the possibility to correct functional prolidase defects by promoting enzyme structure stabilization/recovery was considered. In order to rescue prolidase activity *in vitro* we focused on supporting its folding process by inducing expression of intracellular chaperones. Such an approach brought to 20–40% recovery of the prolidase enzymatic activity in fibroblasts bearing the mutated enzymes; the rescue was mutation-dependent. These results are an exciting proof-of-principle that bears implications for PD therapy. The administration of existing drugs stimulating the endogenous chaperones, or of chaperone-like molecules, might in fact be considered as a means for rescuing the PD phenotype.

## Supporting Information

Figure S1 **Protein melting temperatures in the absence of the cofactor as revealed by Thermofluor Technology.** The solvatochromic dye SYPRO orange was used as an indicator of protein unfolding (fluorescence excitation λ = 492 nm; fluorescence emission λ = 568 nm).
(TIF)

Figure S2 **Alignment of different prolidase amino acid sequences from the region involved in cofactor binding.** Metal binding residues are indicated by arrows. GenBank® Identifier (gi) numbers of the displayed sequences are as follow: 189842 (*H. sapiens*), 296477856 (*B. Taurus*), 9795244 (*M. musculus*), 160774330 (*D. rerio*), 18977119, (*P. furiosus*), 14590977 (*P. horikoshii OT3*).
(TIF)

Table S1 **Hydrodynamic radiuses of the recombinant prolidase variants observed with Dynamic Light Scattering.** R_h_ values and the percentages of polydispersion are reported as the averages of two independent experiments for each protein sample.
(DOC)
