# Supplementary figures and images for: Kinetic and Structural Evidences on Human Prolidase Pathological Mutants Suggest Strategies for Enzyme Functional Rescue
Source: PLoS One. 2013 Mar 13;8(3):e58792. doi: 10.1371/journal.pone.0058792 (PMC3596340; doi:10.1371/journal.pone.0058792)

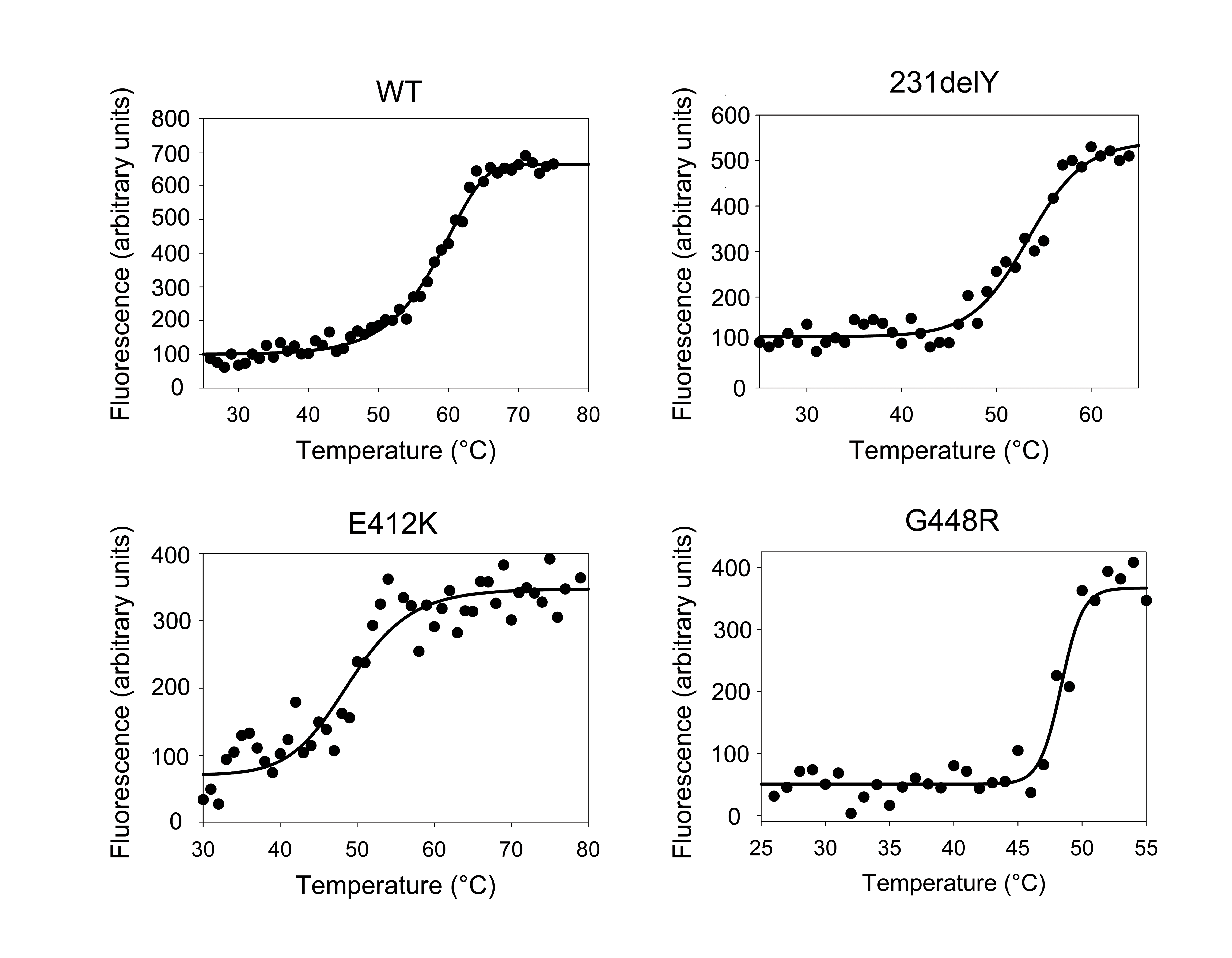

Supplement: Figure S1 — Protein melting temperatures in the absence of the cofactor as revealed by Thermofluor Technology. The solvatochromic dye SYPRO orange was used as an indicator of protein unfolding (fluorescence excitation λ = 492 nm; fluorescence emission λ = 568 nm). (TIF) [file pone.0058792.s001.tif]

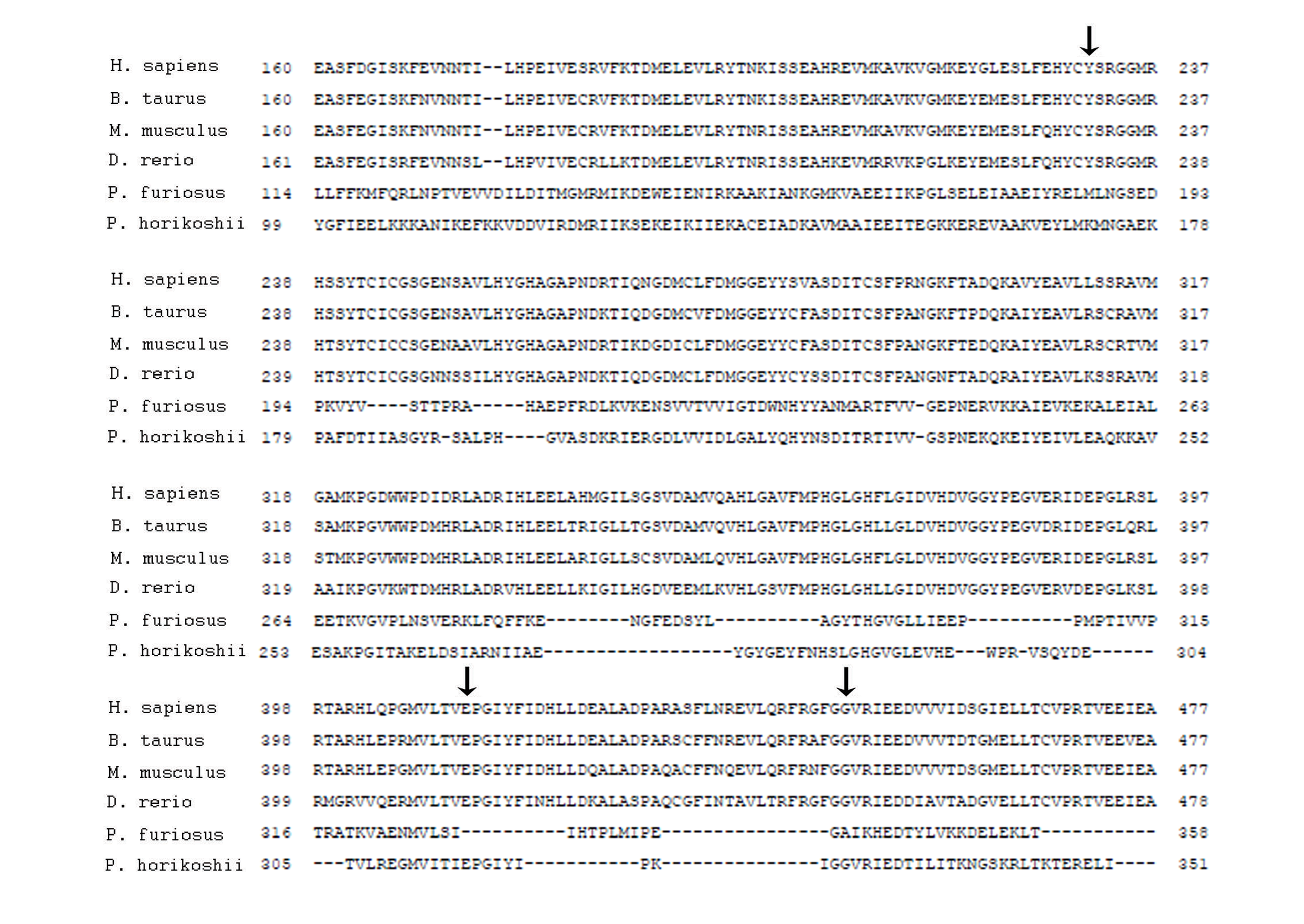

Supplement: Figure S2 — Alignment of different prolidase amino acid sequences from the region involved in cofactor binding. Metal binding residues are indicated by arrows. GenBank® Identifier (gi) numbers of the displayed sequences are as follow: 189842 (H. sapiens), 296477856 (B. Taurus), 9795244 (M. musculus), 160774330 (D. rerio), 18977119, (P. furiosus), 14590977 (P. horikoshii OT3). (TIF) [file pone.0058792.s002.tif]
