# Supplementary material for: Kinetic and Structural Evidences on Human Prolidase Pathological Mutants Suggest Strategies for Enzyme Functional Rescue
Source: PLoS One. 2013 Mar 13;8(3):e58792. doi: 10.1371/journal.pone.0058792 (PMC3596340; doi:10.1371/journal.pone.0058792)
Supplement: Table S1 — Hydrodynamic radiuses of the recombinant prolidase variants observed with Dynamic Light Scattering. Rh values and the percentages of polydispersion are reported as the averages of two independent experiments for each protein sample. (DOC) [file pone.0058792.s003.doc]

**Table S1.** Hydrodynamic radiuses of the recombinant prolidase variants observed with Dynamic Light Scattering. R_h_ values and the percentages of polydispersion are reported as the averages of two independent experiments for each protein sample.

| **hRecProl** | **R_h_ (nm)** | **Polydispersity (%)** |
| --- | --- | --- |
| **WT** | 5.7±0.1 | 22.4±0.8 |
| **231delY** | 5.0±0.2 | 17.0±3.1 |
| **E412K** | 5.25±0.05 | 25.7±0.1 |
| **G448R** | 5.0±0.3 | 28.0±2.6 |
